# Primate richness and abundance is driven by both forest structure and conservation scenario in Costa Rica

**DOI:** 10.1371/journal.pone.0290742

**Published:** 2023-09-13

**Authors:** Christopher Eric Johnson, Kathryn A. Tafoya, Peter Beck, Amy Concilio, Kurt E. White, Rodolfo Quirós, Michael D. Wasserman

**Affiliations:** 1 Department of Anthropology, Indiana University, Bloomington, IN, United States of America; 2 Center for the Integrative Study of Animal Behavior, Indiana University, Bloomington, IN, United States of America; 3 Department of Environmental Science and Policy, St. Edward’s University, Austin, TX, United States of America; 4 Department of Epidemiology and Biostatistics, School of Public Health, Indiana University, Bloomington, IN, United States of America; 5 Las Cruces Research Station, Organization for Tropical Studies, San Vito, Costa Rica; Southeastern Louisiana University, UNITED STATES

## Abstract

Variation in tropical forest management directly affects biodiversity and provisioning of ecosystem services on a global scale, thus it is necessary to compare forests under different conservation approaches such as protected areas, payments for ecosystem services programs (PES), and ecotourism, as well as forests lacking any formal conservation plan. To examine the effectiveness of specific conservation approaches, we examined differences in forest structure and tree recruitment, including canopy cover; canopy height; seedling, sapling, and adult tree density; and average and total diameter at breast height (DBH) across 78 plots in 18 forests across Costa Rica representing protected areas, private forests utilizing PES and/or ecotourism, and private forests not utilizing these economic incentives. The effectiveness of conservation approaches in providing suitable primate habitat was assessed by conducting broad primate census surveys across a subset of eight forests to determine species richness and group encounter rate of three primate species: mantled howler monkey (*Alouatta palliata*), Central American spider monkey (*Ateles geoffroyi*), and the white-faced capuchin monkey (*Cebus imitator*). Only canopy height was significantly different across the three approaches, with protected areas conserving the tallest and likely oldest forests. Canopy height was also significantly associated with the group encounter rate for both mantled howler and spider monkeys, but not for capuchins. Total group encounter rate for all three monkey species combined was higher in incentivized forests than in protected areas, with capuchin and howler monkey group encounter rates driving the trend. Group encounter rate for spider monkeys was higher in protected areas than in incentivized forests. Incentivized conservation (PES and ecotourism) and protected areas are paragons of land management practices that can lead to variation in forest structure across a landscape, which not only protect primate communities, but support the dietary ecologies of sympatric primate species.

## Introduction

Due to the detrimental effects of global change on tropical forests, much attention has been placed on how effective management can protect the ability of tropical forest ecosystems to provide ecosystem services and maintain biodiversity. Recent findings have uncovered that global pervasive shifts in vegetation dynamics undermine forest functioning as old-growth forests with stable dynamics are now less prevalent than younger stands with faster turnover [1]. Given the important role tropical forests play in providing resilience against climate change, effective protection will enhance long-term climate mitigation and adaptation potential [2]. Furthermore, as drivers of global change will likely continue to accelerate shifts in vegetation dynamics over time, it is imperative to uncover specific conservation policies and initiatives that successfully confront and mitigate these shifts to safeguard longstanding sustainable forests [1]. Effective forest conservation strategies are especially critical to human systems to sustain food production, maintain freshwater and forest resources, regulate climate and air quality, and ameliorate infectious diseases [3].

Biodiversity hotspots, such as tropical forest regions, continue to be affected by deforestation and human activity due to the high diversity and quantity of goods and services their ecosystems provide [4]. Like many of these hotspots, Costa Rica experiences ecological pressures from land use practices and deforestation for agriculture, urbanization, and tourism [5]. Costa Rica highlights the need for further understanding the trade-offs between human activity and ecosystem health [6]. As persistent global shifts in vegetation dynamics will ultimately lead to loss in canopy cover and biomass, Costa Rica has implemented multiple conservation initiatives to directly address these concerns [7,8]. Consequently, uncovering how certain policies differentially affect tropical forest structure and biodiversity in a location such as Costa Rica where these strategies have been successfully implemented could reveal specific scenarios applicable to other vulnerable ecosystems on a global scale.

One of the earliest effective practices for land protection in Costa Rica was the National Park System (NPS), which was first implemented in 1969 and by 1996 consisted of 230 protected areas, including 32 national parks, that covered 25–28% of the nation’s land [8,9]. Some of these reserves are managed by nonprofit organizations as research stations, promoting international collaboration on research and education in addition to conservation [8,10]. The most novel of Costa Rica’s approaches is their payments for ecosystem services program (PES), or *pagos por servicios ambientales* (PSA) [11]. This innovative conservation initiative, the first such program in the world, provides funding to landowners that allow land to regenerate naturally and remain undisturbed for “watershed protection, biodiversity conservation, carbon sequestration, and aesthetic values” [11,12]. For example, Costa Rica’s PSA program has led to an overall increase in landscape connectivity across participating properties as well as improved reforestation efforts and enhanced local economic opportunity [13,14]. While there are more lucrative land uses, the PES program has been beneficial for over 587,000 acres of land since it began in 1999 [8,15,16]. The increasing acquisition of public land transformed to national parks along with the reforestation of agricultural land through PES has also led to an increasing amount of ecotourism [11,15]. The NPS program relies on ecotourism to fund the maintenance of the parks and compensate its employees [9]. Revenue from ecotourism activities and services have transformed Costa Rica’s economy with more than two million visits to the country annually [8]. However, many forest fragments across Costa Rica still exist under non-incentivized ownership [16].

To evaluate the effectiveness of these various conservation approaches, on the ground ecological data are needed to examine differences in forest structure and recruitment, which are particularly critical to provisioning of ecosystem services and providing suitable habitat for biodiversity. Forest structure, including canopy height and coverage, and recruitment can be influenced by several factors including the size and age of the forest, edge effects, and elevation [17]. Conservation strategies also influence forest structure, but research on these effects is usually limited to one approach rather than through a comparative analysis. For example, the NPS has been evaluated by comparing how its forest cover compared with surrounding fragments [18]. Deforestation rates within 1-km of these protected areas were similar to rates inside the protected areas, but significant forest loss was present between 1 to 10-km from the parks [19].

Successful conservation strategies that work to enhance forest structure also carry the goal of protecting the biodiversity contained within the forested system to maintain ecosystem functioning [20]. For example, over 65% of primate populations are presently threatened by extinction and primate species make up 25–49% of frugivore biomass in tropical forests, serving as important seed dispersers [21–23]. Given their role in seed dispersal and additional affects from folivory, primates are considered ecosystem engineers [24,25]. Therefore, effective conservation scenarios not only protect forests but also species with critical functional roles such as primates. We previously found that a conservation policy portfolio that included protected areas, ecotourism, and PES in specific regions of Costa Rica led to a decrease in deforestation rates, an increase in community participation, and a complete primate community [26]. However, how differing conservation scenarios across a landscape influence forest structure and recruitment, primate abundance, and their influence on one another remains unclear.

To address the relationships between conservation approaches, forest structure and recruitment, and primate abundance, we examined a series of forests in Costa Rica within (1) protected areas, (2) incentivized approaches (i.e., privately-owned land utilizing ecotourism and/or PES), or (3) privately-owned land without use of conservation incentives. We predicted that 1) the use of incentives would have a significant and positive effect on forest structure and recruitment compared to no use of incentives, 2) protected areas would have the most significant and positive effect on forest structure and recruitment compared to both categories of privately-owned forests, and 3) protected forests would contain the most primate species and have the highest primate abundance.

## Methods

### Study area

We conducted this study in two areas in Costa Rica over the course of 20 weeks (January to March 2017 and 2018). We sampled eighteen forests, with each forest categorized as a protected area (n = 4), a privately-owned forest utilizing incentives (n = 7), or a privately-owned forest not utilizing incentives (n = 7). To determine a site’s category, we gave a survey questionnaire to the local landowner or land manager asking about their participation in PES or ecotourism. Survey questionnaires contained fixed-response questions and were administered orally in Spanish using local translators. Data collection and site selection followed cluster and snowball sampling methods, which utilized expert input from staff at the Organization for Tropical Studies (OTS) for identifying forest fragments for sampling. At the selected sites, we approached surrounding residences to take the survey, and respondents were informed that participation was voluntary and that they may discontinue at any time. Oral consent was obtained before data collection began. The Institutional Review Board at St. Edward’s University and the University of Costa Rica’s Committee on Scientific Ethics preapproved the data collection methods. No personal identification information was collected, and results were coded to ensure anonymity.

**Fig 1:**
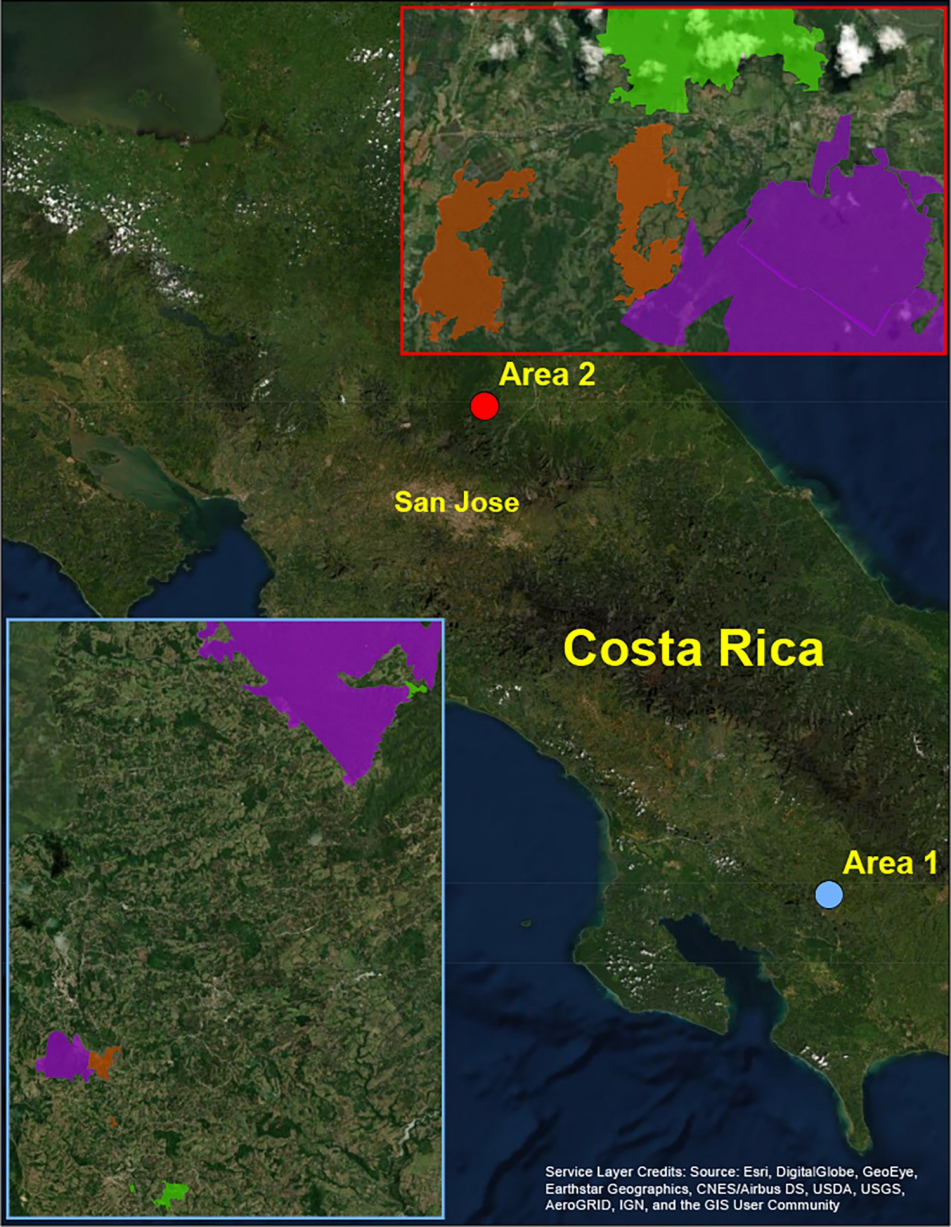
Conservation scenarios across study sites. Three conservation approaches: protected areas (purple), incentivized ownership (orange), and non-incentivized ownership (green) were examined across two areas, Las Cruces (Area 1) and La Selva (Area 2), in Costa Rica, with a total of 18 sites and 78 plots. Reprinted from ArcGIS Pro 3.0 under a CC BY license, with permission from ESRI, original copyright 2023.

Eight study sites were in southern Costa Rica near Las Cruces Biological Station (Fig 1, Area 1) (8° 47’ 7’’ N, 82° 57’ 32’’ W), including the 300-ha fragment owned by OTS, La Amistad International Park, and privately-owned fragments surrounding these two protected areas. This area is characterized by premontane wet forest 1000 to 1400 m above sea level, receives about four meters of rainfall annually, and experiences a distinct dry season from January to March [8]. Ten study sites were located near La Selva Research Station (Fig 1, Area 2) (10° 25′ 19.2” N, 84° 0′ 54″ W), including Braulio Carrillo National Park, the 1,600-ha forest owned by OTS, and privately-owned fragments near these two protected areas. This area is characterized by lowland tropical wet forest around 35 m above sea level in northeastern Costa Rica and receives about four meters of rainfall annually [8].

### Forest structure and location

Nested plots were constructed to sample the plant community by life stage in each forest fragment [27]. We counted all adult woody individuals inside the outer circular plot with an area of 150 m^2^, all saplings inside an inner subplot with an area of 75 m^2^, and all seedlings inside three 1 m^2^ plots randomly placed within the sapling subplot. At least three nested plots were placed at each site: along the edge (0–500 m from forest boundary), in the middle of the forest (> 500 m from forest boundary), and near an adult fig tree (*Ficus spp*.). A fig plot was targeted due to their role as a keystone species and potential target for restoration [28–30]. The number of replicates at each study site depended on the accessibility, topography, and the size of the forest. Seedlings were classified as having a DBH (diameter breast height = 1.3 m) of 0–0.5 cm, saplings were 0.5–10 cm, and adults were > 10 cm [31–34]. We used a Vernier caliper with a set diameter range to count seedling and sapling individuals and a DBH tape to measure each adult individual tree. Raw DBH values were used to calculate cumulative DBH and average DBH for each nested plot.

In the center of each nested plot, we used a convex spherical densiometer to calculate percent canopy cover. Four measurements were taken at each ordinal direction and then averaged to be representative of the canopy directly above the nested plot. Canopy height was measured at the edge of each nested plot using the standard method with a Suunto Clinometer PM-5 [35]. By using the diameter of the nested plot and a consistent eye height value of 1.66m, multiple measurements were taken along the perimeter of the nested plot to calculate the average height (m) for each nested plot.

For each nested plot, we calculated canopy cover (%), canopy height (m), seedling density (# seedlings/m^2^), sapling density (# saplings/m^2^), adult density (# adults/m^2^), total DBH, and average DBH. A GPS point was taken in the center of each nested plot using a Garmin (Oregon 600) GPS unit, which recorded the plot’s latitude, longitude, and elevation (m). By mapping the nested plots in ESRI ArcGIS Pro 3.0 on an aerial image of each study area, polygons were created to represent the physical boundary based on the ecological boundary of each study site. These polygons were created by drawing lines parallel to the forest edge observed in the aerial imagery. Using these polygons, we calculated the total size (hectares) for each site, as well as the distance (m) between each nested plot and the nearest protected area.

All analyses for descriptive statistics were completed in SAS statistical software (version 9.4, SAS Institute, Inc., Cary, North Carolina). Using one-way ANOVA, we tested for significant differences for each variable across the three conservation scenarios. To control for latitude, longitude, and elevation and determine which specific conservation scenarios differed from one another, we employed multilevel modeling in R using linear mixed effects [36]. By nesting plots within sites and sites within areas as random effects, we accounted for any spatial autocorrelation that would have violated the assumption of independence in sampling units. Two separate linear mixed effects models were used to evaluate the association between each ownership category and forest structure characteristics, one with protected areas as the reference and the other with no incentives as the reference.

### Primate group encounter rate and species richness

Primate censuses were conducted at a subset of 8 forested sites to compare the abundance of three species: mantled howler monkey (*Alouatta palliata*), Central American spider monkey (*Ateles geoffroyi*), and white-faced capuchin monkeys (*Cebus imitator*) *across* protected areas (n = 3) and incentivized conservation fragments (n = 5). Broad survey methods are suitable for collecting comparative data on the presence and species richness for a large geographic area in a relatively short amount of time across several sites [26,37]. Surveys were conducted along 1 km segments of trail that were each surveyed twice, once in the morning and once in the afternoon between 08:00–17:00 h across ten weeks in 2019. A total of 138 1 km line transects were surveyed twice and transects were walked at a slow and consistent pace of 1km/h, stopping every 50 meters for 1 min to listen for primate activity. Upon encountering a group in a line transect, time, group count, age and sex composition, activity, and location data were collected in under 5 min to minimize disturbance. Primate group encounter rate was calculated by dividing total primate groups observed per forest by total kilometers walked, and primate species richness was recorded as percent species observed of expected species in each forest [26]. In testing for associations between forest structure and primate group encounter rate, separate generalized linear regression models were used to evaluate the association between forest variables (canopy cover (%), canopy height (m), number of seedlings/m^2^, size (ha), and distance to protected area (m)), conservation scenario, and primate group encounter rate, for each monkey species and for the primate community.

## Results

Across 78 nested plots in 18 forests, a total of 689 seedlings, 3254 saplings, and 609 adult trees were measured. When comparing recruitment across the different conservation approaches, the average number of seedlings per m^2^ was 7.53 (+/- 4.53 SD) for protected areas, 9.51 (+/- 6.17 SD) for non-incentivized forests, and 10.53 (+/- 6.62 SD) for incentivized forests. For forest structure, the average canopy height was lowest in non-incentivized forests at 11.59m (+/- 3.23 SD), 13.56m (+/- 1.90 SD) for incentivized forests, and highest in protected areas at 14.37m (+/- 2.18 SD) (Table 1). Similarly, canopy cover was lowest for non-incentivized forests at 77.71% (+/- 9.03 SD), 81.47% (+/- 5.42 SD) for incentivized forests, and highest for protected areas at 82.72% (+/- 5.10 SD). Also, an increase in the average size of the forest followed a similar trend with the smallest for non-incentivized forests at 233.26 ha (+/- 559.41 SD), 1046.15 ha (+/-874.40 SD) for incentivized forests, and largest for protected areas at 55,831.86 ha (+/- 81,473.81).

**Table 1:** One-way ANOVAs comparing forest composition variables across three different conservation scenarios in Costa Rica.

	Conservation Scenario						
Characteristic	None		Protected Areas^1^		Incentivized Conservation		P-Value
Number of Sites	7		4		7		-
Number of Plots	15		39		24		-
Cover (%)	77.71	(9.03)	82.72	(5.10)	81.47	(5.42)	0.13
**Height (m)**	**11.59**	**(3.23)**	**14.37**	**(2.18)**	**13.56**	**(1.90)**	**0.001**
**Elevation (m)**	**725.92**	**(545.58)**	**887.12**	**(765.55)**	**382.39**	**(452.97)**	**0.01**
**Latitude**	**9.43**	**(0.83)**	**9.50**	**(0.76)**	**9.96**	**(0.77)**	**0.04**
**Longitude**	**-83.33**	**(0.54)**	**-83.39**	**(0.54)**	**-83.74**	**(0.53)**	**0.02**
**Size (Hectares)**	**233.26**	**(559.41)**	**55831.86**	**(81473.81)**	**1046.15**	**(874.40)**	**<0.0001**
**Distance to Protected Area (m)**	**3695.00**	**(2740.76)**	**0.00**	**(0.00)**	**1313.93**	**(2131.49)**	**0.01**
Number of Seedlings/m^2^	9.51	(6.17)	7.53	(4.53)	10.53	(6.62)	0.11
Number of Saplings/m^2^	0.53	(0.37)	0.58	(0.35)	0.54	(0.23)	0.79
Number of Adults/m^2^	0.05	(0.02)	0.05	(0.02)	0.05	(0.02)	0.55
Number of Adults/plots	8.07	(3.08)	7.29	(2.82)	8.03	(2.63)	0.55
Total DBH (cm)	245.11	(103.77)	244.82	(103.27)	231.00	(93.47)	0.85
Average DBH (cm)	30.91	(10.54)	32.05	(14.05)	33.09	(12.39)	0.88

^1^Because of laws surrounding the ownership and management of national parks and research stations, both are considered protected areas.

*Significant values are bolded.

All values are mean and standard deviation or frequency and percent.

**Fig 2:**
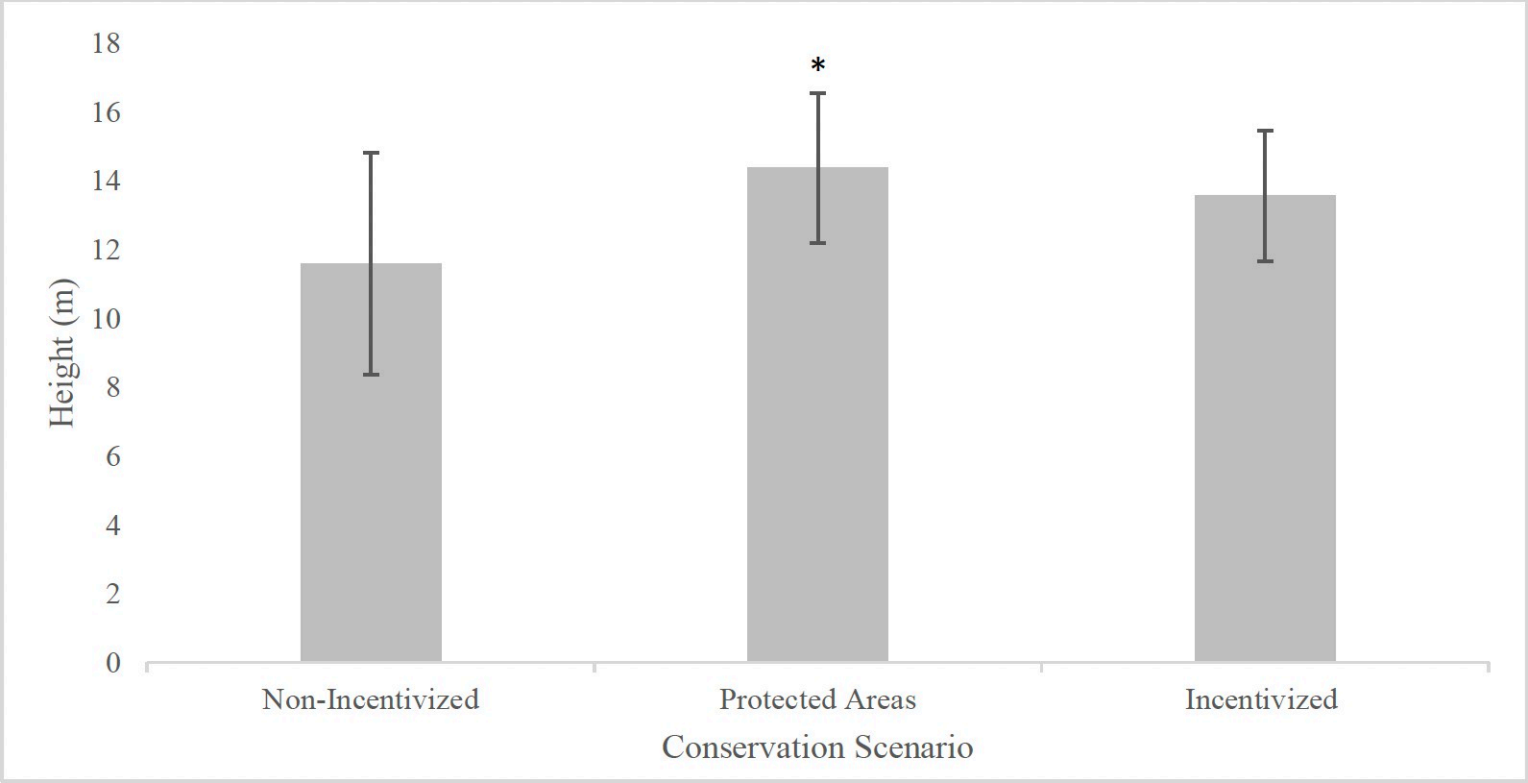
Canopy height and conservation scenarios. Differences in canopy height across forests in protected areas, privately-owned forests utilizing conservation incentives, and privately-owned forests not utilizing incentives. Protected areas had a significantly taller canopy height (m) than both incentivized and non-incentivized privately-owned forests.

One-way ANOVA tests comparing each variable across the three conservation approaches indicated that latitude, longitude, elevation, size, distance to a protected area, and canopy height displayed significant differences (*p* < 0.05, Table 1), while canopy cover, number of adult trees per area, measures of DBH, and estimates of recruitment were not significantly different (*p* > 0.05). Linear mixed effects models controlled for latitude, longitude, elevation, and spatial configuration of sites and plots to minimize the confounding influence of natural ecological variation and spatial autocorrelation on forest structure. When compared to forests without use of conservation, protected areas had significantly higher canopy height (*p* = 0.01, Fig 2) and forests utilizing conservation incentives (PES or ecotourism) had shorter distance to protected areas (*p* = 0.02).

**Fig 3:**
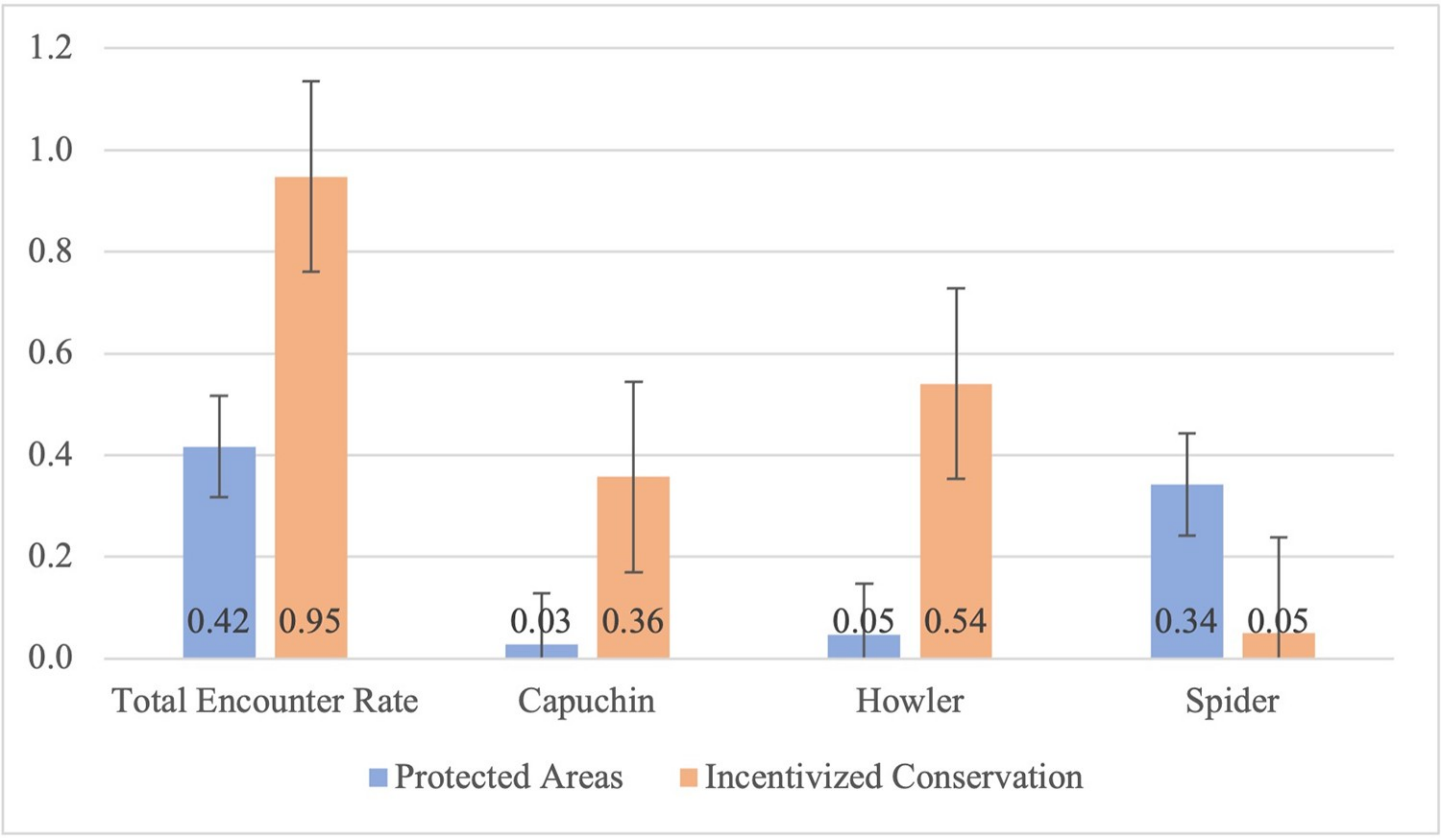
Conservation scenarios compared to primate group encounter rate for the primate community and each monkey species. Primate group encounter rate (group/hour) for the primate community (total encounter rate) was higher for incentivized conservation scenarios than protected areas, and both capuchin and mantled howler monkeys had higher group encounter rates in forests with incentivized conservation scenarios, and their group encounter rate was higher than that of spider monkeys. The group encounter rate for spider monkeys was higher for protected areas than for forests with incentivized conservation scenarios.

In comparing associations between forest structure and the primate community, significant variables from the forest structure and conservation scenario analyses were tested against outcomes of primate group encounter rate in separate generalized linear regression models. Forests with either conservation scenario (incentivized conservation or protected areas) contained all three monkey species. Incentivized conservation scenarios were associated with a higher total primate group encounter rate (*p* = 0.0001, Table 2) and a higher group encounter rate for capuchin (*p* = <0.0001) and howler monkeys (*p* = 0.0003) compared to protected areas (Fig 3). Spider monkey group encounter rate was highest in protected areas (*p* = 0.096), but this relationship was not significant. Canopy height (*p* = 0.0096) and forest size (*p* = <0.0001) was significantly associated with spider group encounter rate, while canopy height (*p* = 0.048) was associated with group encounter rate for howler monkeys (Table 2).

**Table 2:** Comparisons between conservation scenarios, forest composition variables, and primate group encounter rate. Beta coefficients with 95% confidence intervals from generalized linear regression for primate encounter rate according to forest variables and conservation scenario with protected areas as a reference. Significant values (*p* < 0.05) are bolded.

	Total Encounter Rate			Capuchin Encounter Rate	
		β	P-Value	β	P-Value
Cons. Scenario Protected Areas		Ref.	NA	Ref.	NA
Incentivized Conservation		0.841 (0.441, 1.241)	**0.0001**	0.347 (0.193, 0.500)	**<0.0001**
Cover (%)		-0.001 (-0.032, 0.030)	0.953	0.010 (-0.002, 0.022)	0.105
Height (m)		0.052 (-0.021, 0.125)	0.1581	-0.022 (-0.050, 0.006)	0.121
Number of seedlings/m^2^		0.015 (-0.017, 0.046)	0.347	0.003 (-0.009, 0.015)	0.594
Size (ha)		0.000002 (-0.0000003, 0.0000046)	0.082	0.00000002 (-0.00000091, 0.00000095)	0.965
Distance to Protected Area (m)		-0.000012 (-0.000130, 0.000106)	0.8395	-0.000025 (-0.000071, 0.00002)	0.2651
		Howler Encounter Rate		Spider Encounter Rate	
		β	P-Value	β	P-Value
Cons. Scenario Protected Areas		Ref.	NA	Ref.	NA
Incentivized Conservation		0.566 (0.276, 0.854)	**0.0003**	-0.071 (-0.156, 0.013)	0.096
Cover (%)		-0.008 (-0.030, 0.015)	0.488	-0.0029 (-0.0095, 0.0037)	0.3758
Height (m)		0.053 (0.0005, 0.106)	**0.048**	0.021 (0.0053, 0.0363)	**0.0096**
Number of seedlings/m^2^		0.011 (-0.011, 0.034)	0.3167	0.0002 (-0.0065, 0.0069)	0.956
Size (ha)		-0.0000009 (-0.0000027, 0.0000008)	0.2958	0.000003 (0.0000025, 0.0000036)	**<0.0001**
Distance to Protected Area (m)		0.000009 (-0.000076, 0.000095)	0.8298	0.000004 (-0.000021, 0.0000029)	0.7311

## Discussion

Understanding the influence of conservation approaches and incentives on tropical forest structure and recruitment is critical to the protection of biodiversity and ecosystem services. Here, we utilized on the ground data analyzed with multilevel mixed modelling to evaluate how three conservation approaches (protected areas, privately-owned forest fragments with owners utilizing ecotourism or PES, and privately-owned forest fragments with owners not using incentives) influence forest structure and recruitment. As we predicted, protected areas were most effective given they had the tallest canopy height. Although we did not detect a significant difference in structure or recruitment between incentivized and non-incentivized privately-owned forests, incentivized sites were closer to protected areas and larger in area. Counter to our predictions, we did not detect any significant differences in our measurements of recruitment across all three approaches.

Our results also indicate that variation in conservation scenario not only influences forest structure, but both conservation scenario and forest structure influence the primate community. Unexpectedly, fragments with incentivized conservation scenarios had a higher total primate group encounter rate than protected areas driven by the increased abundance of capuchins and howler monkeys in those fragments. Howler monkeys were also more abundant in the taller forests, while spider monkeys were more abundant in the taller and larger forests. Given that the mantled howler monkey and the white-faced capuchin monkey are considered vulnerable by the International Union for Conservation of Nature (IUCN) and the Geoffrey’s spider monkey is endangered [38], understanding why these variables predicted their abundance can provide key insights into their conservation.

Differences in diet likely explain our findings. For example, howlers’ more flexible frugivore-folivore diet enables them to persist in fragmented and anthropogenic landscapes [39,40], although they likely still require tall enough trees to meet this nutrient demand through high quality young leaves and fruit [41,42]. Capuchin monkeys can likely persist in relatively young and human-modified landscapes due to their diet characterized as highly omnivorous and opportunistic [43]. They are also known to crop-raid, consuming domestic banana (*Musa acuminata*), mango (*Mangifera spp*.), palm fruits, and coconut (*Cocos nucifera*), thus making fragments suitable habitat when crops are nearby [44]. Alternatively, spider monkeys’ highly frugivorous diet leading to a larger home range and a preference for a high canopy restricts them to larger and taller forests with more fruit availability, such as those we found in protected areas [38,42,45]. Overall, variation in forest structure across landscapes employing different conservation scenarios appears to be crucial for protecting the primate community given differences in dietary niche across the three species.

Effort should be made to limit the occurrence of major disturbance events to ensure continued progression of shorter, younger privately-owned fragments towards the taller old-growth stage given its importance to biodiversity, ecosystem services, and carbon sequestration [2]. Such effort will depend on the conservation approach and be context-dependent to each site, but it includes limiting tourist disturbance at ecotourism sites, limiting unsustainable resource extraction from PES sites, promoting use of incentives at sites not currently using them, and maintaining and even expanding current protected areas. Such effort is even more critical moving forward, as the observed increase of secondary forests throughout Costa Rica since the implementation of multiple conservation approaches indicates that patches of younger forest are likely to become connected to older forests if these different approaches are coordinated in their goals to maintain, regrow, and connect forests. For example, in a case study examining land cover dynamics in the Osa Peninsula of Costa Rica between 1985 and 2009, patches of secondary forest increased in number and size, enhancing the connectivity between different conservation areas in the area [46]. A similar trend was observed in other parts of the country, such as Braulio Carrillo National Park, where protected areas have become connected to neighboring fragments through changes in land acquisition and through forest management of the fragments [18,47,48].

Our results further support the importance of this coordinated effort, as privately-owned forests with incentives were closer to protected areas and larger in size than non-incentivized forests. Mutual ecological and socio-economic benefits are likely to occur when promoting connection of these sites to protected areas. Although conservation of tropical forests in Costa Rica and elsewhere greatly depends on protected areas, all three approaches (i.e. protected, incentivized, and non-incentivized) have potential to conserve forests across successional stages and enhance the distribution and connectivity of old-growth forests when managed in a collective, landscape scale conservation portfolio.

All three of these sympatric primate species in Costa Rica have exhibited dietary and behavioral flexibility across fragmented and human-modified forest patches with differing land use types, contributing to their survival and success in areas of heightened anthropogenic disturbances [38,40,44]. Forest management and conservation strategies that promote matrix habitats, that contain a mixture of native fruit trees and some cultivated ones, between agriculture sites and forests enhance primate habitat suitability and spatial connectivity in Costa Rica [47,48]. Employing matrix habitats between agriculture sites and forests benefits primate communities and enhances forest connectivity; food production systems also benefit by having crop-raiding curbed due to feeding in matrix habitats and diminishing the motivation to further deforest areas to meet agricultural demands [49]. Including multiple sympatric primate species in future community-wide analyses evaluating the effectiveness of a seed-dispersal network could reveal pathways that enhance reforestation in tracing relationships from primate feeding behaviors, food production systems, and tropical forest composition.

As the sites examined in this study were based on volunteer participation, the representation of the landscape of each area was spatially limited. Furthermore, while plots were placed randomly within a site to indicate the overall forest structure, the size of plots are relatively small compared to the size of the forest, especially for the protected areas and largest fragments. Although we attempted to increase the number of plots in larger forests, ultimately the number of plots within a site depended on accessibility. Thus, the results might be skewed by sampling effort and biased towards more accessible forests (e.g., greater representation in forests and areas of forests that were more accessible). Adding more plots within sites and including more sites across a study area in future studies would address these limitations and improve the study design to represent the mosaic landscapes of these two areas more accurately.

Costa Rica has a unique opportunity to continue as a global leader in the conservation of tropical forests and biodiversity by creating a network of privately-owned green highways linking their protected area old-growth forests. The country has been engaged with this work since the 1990s as they have established 44 ecological corridors that cover 38% of their territory [50]. While protected areas were critical to protecting the tallest and likely oldest forests, incentivized and non-incentivized forests shared many similar characteristics to protected forests, including similar recruitment and enhanced primate habitat potential. Given enough time without disturbance, it is likely that they will grow to obtain similar canopy heights.

Overall, our study documented relationships between conservation scenarios, forest structure, and a primate community in Costa Rica, with conservation scenario and forest structure both influencing primate group encounter rate. Protected areas had a higher canopy height than incentivized and non-incentivized forests, while incentivized forests had higher group encounter rates than protected areas for all three species combined, mantled howler, and capuchin monkeys. Spider and howler monkey group encounter rate also increased with forest height. Thus, a mixed approach to the conservation of tropical forests that includes protected areas, PES, and ecotourism will lead to the greatest protection of all three primates while also protecting the tallest forests.
